# Chemical and Rheological Modifications of White Sorghum Flour by Physical Treatments with Possible Implications for Health

**DOI:** 10.3390/molecules31060940

**Published:** 2026-03-11

**Authors:** Ana Batariuc, Mădălina Ungureanu-Iuga, Anca Becze, Lacrimioara Senila, Claudiu Cobuz, Silvia Mironeasa

**Affiliations:** 1 Sanitary, Veterinary and Food Safety Directorate of Suceava, 2nd, Scurta Street, 720223 Suceava, Romania; ana.batariuc@usm.ro; 2 Faculty of Food Engineering, Ștefan cel Mare University of Suceava, 13 Universitatii Str., 720229 Suceava, Romania; silviam@fia.usv.ro; 3 Institute of Advanced Studies, Integrated Research, Development and Innovation Center for Advanced Materials, Nanotechnologies and Distributed Manufacturing and Control Systems (MANSiD), Ștefan cel Mare University of Suceava, 13 Universităţii Street, 720229 Suceava, Romania; 4 Mountain Economy Center (CE-MONT), National Institute of Economic Research (INCE), Romanian Academy, 49 Petreni Street, 725700 Vatra Dornei, Romania; 5 Research Institute for Analytical Instrumentation Subsidiary, National Institute for Research and Development of Optoelectronics Bucharest INOE 2000, 67 Donath Street, 400293 Cluj-Napoca, Romania; anca.naghiu@icia.ro (A.B.); lacri.senila@icia.ro (L.S.); 6 Faculty of Medicine and Biological Sciences, Stefan cel Mare University of Suceava, 13th Universitatii Street, 720229 Suceava, Romania; claudiu.cobuz@usm.ro

**Keywords:** dry heat treatment, particle size, nutritional profile, dough rheology, fatty acid profile, amino acids

## Abstract

This paper aimed to investigate the impact of dry heat treatment and fractionation on white sorghum grain’s chemical and rheological properties. For this, dry heat treatment was applied to sorghum grains of different granulations, integral (I), large (L > 300 μm), medium (200 μm < M < 250 μm), and small (S < 200 μm), at corresponding temperatures of 144 °C, 132 °C (M), and 121 °C (S). The content of amino acids, fatty acids, minerals, and volatile compounds was determined in sorghum flours, along with the dynamic rheological behavior of sorghum dough. The results indicated that dry heat treatment increased mono and polyunsaturated fatty acid content, and decreased lysine, isoleucine, and glutamic acid contents. Significant differences (*p* < 0.05) in amino acid and fatty acid profiles were observed between fractions. Generally, Ca and Na increased after dry heat treatment of sorghum grains, while Fe, Zn, and Cu decreased, except in the M particle size sample. The optimal fraction M is distinguished by an increase in Fe, Zn and Cu content compared to the control. Volatile compounds were affected by both fractionation and dry heat treatment, with samples with S particle size possessing a distinct volatile profile. Dry heat treatment produced a stiffer, less deformable dough, maintaining elastic dominance and slightly reducing the peak gelatinization temperature. Particle size reduction led to dough strengthening and an increase in elastic and viscous moduli. The combined use of fractionation and dry heat treatment permits precise control of sorghum’s nutritional and rheological properties.

## 1. Introduction

Sorghum is a major global cereal crop, ranking as the fifth largest after rice, maize, wheat, and barley [1]. It is particularly vital in the warm and arid regions of the world due to its high resistance to drought and heat, serving as a staple food providing energy, protein, vitamins, and other essential nutrients to millions of people. Sorghum is especially significant as a gluten-free cereal, making it a valuable ingredient for individuals with celiac disease or wheat allergy [1,2]. The sorghum kernel is structurally complex, consisting mainly of the endosperm (~84%), germ (~10%), and pericarp (bran, ~6%) [3]. This structure dictates the distribution of nutrients [4].

The performance and functional quality of sorghum (*Sorghum bicolor* (L.) Moench) are strongly influenced by the choice of variety, local growing conditions, and prevailing weather. Cultivar selection determines kernel color, starch composition, protein content, and resistance to biotic and abiotic stresses, all of which impact both nutritional and technological properties [5]. Environmental factors, including temperature, rainfall, soil type, and seasonal variability, affect grain development, starch granule morphology, and protein–starch interactions [5], ultimately influencing flour functionality and dough rheology. For example, warm temperatures during grain filling enhance starch deposition, while water limitation can reduce protein quality and modify amino acid profiles [6]. Consequently, understanding the interplay between genotype and environment is critical when evaluating sorghum for gluten-free applications, as both nutritional composition and functional properties are shaped not only by thermal processing but also by the cultivar and its growth environment. Studies in temperate regions, such as the Secuieni Agricultural Research and Development Station in Neamț, Romania, highlight that moderate precipitation, warm summers, and cold winters create conditions that challenge crop adaptation but allow assessment of drought-tolerant sorghum varieties for food applications [7].

Sorghum protein quality is often considered poor because it is deficient in critical essential amino acids, most notably lysine, as well as threonine and total sulfur amino acids (methionine and cysteine) [8]. However, it is rich in glutamic acid, proline, and leucine [8,9]. The dominant storage proteins are kafirins (prolamins), which are highly hydrophobic and tend to form disulfide cross-links due to their high cysteine content, contributing to low protein digestibility [10]. Sorghum lipids primarily consist of linoleic and oleic acids [2]. The lipid content is mostly concentrated in the germ and pericarp [11]. The presence of unsaturated fatty acids, while nutritionally superior, makes sorghum flour susceptible to rancidity and the development of off-flavor when stored, especially after milling [11]. Sorghum grains are sources of minerals, including phosphorus (P), potassium (K), magnesium (Mg), sodium (Na), calcium (Ca), iron (Fe), and zinc (Zn) [12]. The germ fraction is also rich in minerals [4].

Heat treatment is critical for improving the storage stability of sorghum flour. The process works by inactivating lipid-degrading enzymes, primarily lipase, which are released during milling [1,11]. Lipase degrades triglycerides, resulting in increased free fatty acids and the onset of hydrolytic rancidity and off-flavor [11]. Heat processing significantly impacts sorghum proteins. Cooking and wet heat processing conditions tend to reduce protein digestibility due to the formation of disulfide cross-links involving the cysteine-rich β- and γ-kafirins [10]. These cross-links encapsulate starch granules, inhibiting enzyme access. Conversely, studies on specific cooking/processing methods have shown that cooking raw sorghum flour can significantly increase the amino acid content, including lysine [9]. Dry heat treatments (roasting) may reduce crude protein content due to thermal degradation or loss of volatile nitrogenous compounds [13]. Thermal processing (such as cooking or dry heat) generally leads to a decrease in mineral content (macro- and microelements) due to leaching [12]. However, processing techniques often reduce anti-nutritional factors (like phytates), which in turn significantly enhance the bioavailability and extractability of minerals such as iron and zinc [9,12].

Particle size resulting from milling significantly influences the flour’s functional properties, digestion kinetics, and chemical composition because it determines the proportion of anatomical parts (pericarp, germ, endosperm) included. An increase in particle size (i.e., coarser flour) corresponds to an increase in protein, carbohydrate, and fiber content in dry-heat-treated red sorghum flour. Conversely, fat, ash, and moisture content decreased as particle size increased, meaning finer flour tends to concentrate the oil-rich germ fraction and ash [2]. For instance, fine milling (dehulling) of Soriz grain reduced the total amount of essential amino acids compared to the native grain [14].

Particle size profoundly affects dough strength and flow characteristics due to differences in hydration behavior and component distribution [4]. Generally, smaller sorghum flour particles exhibit greater water absorption [4]. However, depending on the composition, decreasing particle size may lead to complex outcomes: in some milled cereal flours, viscosity increases with larger particle size [13], while in other composite systems (e.g., wheat bran), certain strength parameters measured under external forces remain independent of particle size, suggesting that only strongly bound water contributes to the overall strength/viscosity under stress [15]. Consequently, controlling particle size is essential for manipulating the mechanical strength (resistance to deformation) and processing quality of sorghum-based products.

Understanding how thermal processing affects the functionality of sorghum, a gluten-free cereal with growing importance in sustainable food systems, is essential for improving its technological performance in dough and baked products. While previous research has examined the effects of hydrothermal or extrusion treatments on sorghum starch, limited information exists on how dry heat treatment influences the chemical composition and rheological behavior of sorghum flours, particularly across different granule size fractions. This study aimed to combine fractionation by particle size with controlled dry heat treatment to elucidate how variations in grain microstructure and protein–starch interactions alter amino acid, fatty acid, mineral, and volatile profiles, as well as dough viscoelasticity. By linking thermal modification and granule size to changes in functional and structural properties, this work provides new insights into tailoring sorghum flours for enhanced dough strength, elasticity, and nutritional value, filling a key gap in cereal chemistry and gluten-free formulation research. Our previous works [2,16,17] were largely focused on proximate composition and basic functional properties, with little emphasis on dough viscoelasticity, and comprehensive nutritional and volatile profiling. The present study advances the field by providing an integrated and mechanistic evaluation of how controlled particle size and dry heat temperature jointly modulate both the rheological performance and compositional quality of sorghum flours.

## 2. Results and Discussion

### 2.1. Amino Acid Profile

**Figure 1:**
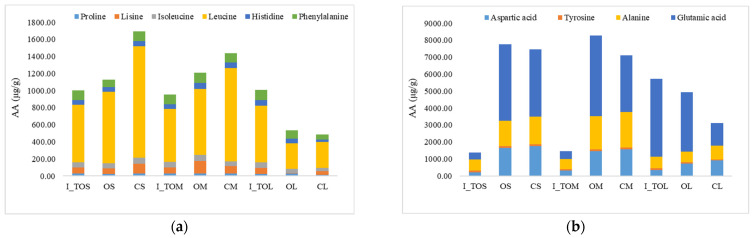
Essential (**a**) and non-essential (**b**) amino acid (AA) content of sorghum flours: integral white sorghum flour treated at the optimal temperature specific to each fraction, optimal fractions, and control fractions: I_TOS—integral sorghum flour treated at 121 °C; I_TOM—integral sorghum flour treated at 132 °C; I_TOL—integral sorghum flour treated at 140 °C; CS—untreated control sample with S particle size (<200 μm); OS—optimal treated sample at 121 °C with S particle size (<200 μm); CM—untreated control sample with M particle size (200 μm < M < 250 μm); OM—optimal treated sample at 132 °C with M particle size (200 μm < M < 250 μm); CL—untreated control sample with L particle size (>300 μm); and OL—optimal treated sample at 140 °C with L particle size (>300 μm).

The amino acid profile of the white sorghum flour fractions indicated the presence of essential amino acids, proline, lysine, isoleucine, leucine, phenylalanine, and histidine, as well as non-essential amino acids such as aspartic acid, tyrosine, and glutamic acid. The content of essential amino acids is presented in Figure 1a. From a clinical nutrition perspective, this profile is relevant because leucine and isoleucine are branched-chain amino acids (BCAAs) that participate in postprandial metabolic signaling; leucine has been proposed as a key “trigger” for the stimulation of muscle protein synthesis (via mTORC1-related pathways), with greater practical relevance in older adults and in contexts of anabolic resistance, although effects depend on adequate total protein/EAA supply and the food matrix [18]. Conversely, higher habitual BCAA exposure has been associated with adverse cardiometabolic phenotypes (e.g., insulin resistance/NAFLD) in some observational datasets, so any “BCAA-rich” positioning of cereal fractions should be interpreted in a whole-diet context, rather than as an isolated amino acid effect [19,20].

All samples showed a high leucine content, which decreased with decreasing particle size. For the S and M fractions, the leucine content was higher in the optimal fractions than in the integral flour, but lower than in the control fractions S and M, while for the L fraction, no significant differences were observed between the optimal and control fractions. The lysine and isoleucine contents decreased after applying the dry heat treatment, except for the optimal M fraction. Similar results were reported by Mohapatra et al. [21], who found a decrease in essential amino acid content after steam treatment, probably due to protein denaturation and/or the formation of bonds with phenolic compounds. Importantly, lysine is typically the first limiting indispensable amino acid in sorghum-based diets; human indicator amino acid oxidation data suggest that lysine bioavailability from cooked sorghum can be high, yet the absolute lysine content remains limiting, supporting practical strategies such as protein complementation with lysine-rich legumes when designing health-oriented sorghum foods [22]. The intensification of Maillard reactions could also explain the reduction in the content of some amino acids. In particular, non-enzymatic glycation during heating can make lysine nutritionally “unavailable” (loss of reactive lysine) even when total lysine is still detected analytically, thereby lowering true protein quality; Maillard chemistry in cereal matrices is also discussed in relation to broader health-relevant endpoints (e.g., formation of advanced Maillard products) [18,23].

Regarding particle size differences, it has been shown that integral sorghum grains and the endosperm are richer in glutamic acid, proline, alanine, and leucine, whereas the germ and pericarp are richer in glycine, lysine, and arginine [24]. This supports the idea that fractionation can shift amino acid limiting patterns, but also that outer-layer enrichment may increase exposure to phenolics/tannins that can complex with proteins and reduce digestibility [20,25]. It can therefore be concluded that white sorghum is a source of branched-chain amino acids (leucine and isoleucine), similar to soriz [14].

The results obtained for the content of non-essential amino acids indicate remarkable variations depending on the type of sample (Figure 1b). These highlight that the heat treatment and fractionation determined a different content in non-essential amino acids depending on the optimal temperature applied to white sorghum grains and the optimal fraction of white sorghum flour. A high content of glutamic acid was obtained in the OM compared to the CM, an amino acid that is found in a low proportion in integral white sorghum flour treated at the optimal temperature for the M fraction (I_TOM). The content of alanine and aspartic acid was lower in the integral samples compared to the treated and untreated fractions. A decrease in the content of glutamic acid is noted after the application of dry heat treatment. Mokrane et al. [26] also reported a high content of glutamic acid in sorghum flour. The increase in the content of some amino acids could be due to the reduction in moisture content during heat treatment, which could increase the concentration of constituents in the flour [9]. According to a previous study [27], heat treatment can affect amino acids differently depending on particle size, as finely ground soybean meal exposed to thermal processing exhibited greater heat damage to amino acids than coarser particles, indicating the increased susceptibility of smaller particles to thermal effects. In roasted soybean, increasing roasting temperature and time reduced soluble protein, and true protein fractions changed differently with particle size, with smaller particles showing increased intermediate true protein fractions under heat, while larger particles showed different responses, indicating that heat and particle size interactively influence protein characteristics [28] and consequently the amino acid profile.

### 2.2. Fatty Acid Profile

**Figure 2:**
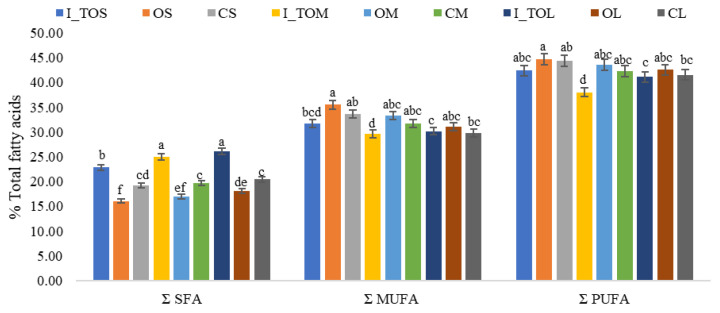
Total saturated (SFA), monounsaturated (MUFA) and polyunsaturated (PUFA) fatty acids in integral white sorghum flour treated at the optimal fraction-specific temperature, in the optimal, and control fractions: I_TOS—integral sorghum flour treated at 121 °C; I_TOM—integral sorghum flour treated at 132 °C; I_TOL—integral sorghum flour treated at 140 °C; CS—untreated control sample with S particle size (<200 μm); OS—optimal treated sample at 121 °C with S particle size (<200 μm); CM—untreated control sample with M particle size (200 μm < M < 250 μm); OM—optimal treated sample at 132 °C with M particle size (200 μm < M < 250 μm); CL—untreated control sample with L particle size (>300 μm); and OL—optimal treated sample at 140 °C with L particle size (>300 μm).

The results obtained for the fatty acid content in thermally treated white sorghum grains at the optimal temperature determined for each fraction (I_TOS, I_TOM, and I_TOL), in the optimal white sorghum flour fractions (OS, OM, and OL), and in the control fractions (CS, CM, and CL) indicate remarkable variations depending on the type of sample (Figure 2, Table 1).

The polyunsaturated (PUFA), monounsaturated (MUFA), and saturated fatty acids (SFA) present in the analyzed samples varied in the following order: PUFA > MUFA > SFA. The heat treatment led to an increase in PUFA and MUFA and a decrease in SFA in the optimal fractions compared to the control fractions. Among the optimal samples, fraction S (OS) stood out for its high PUFA and MUFA content and low SFA content. Particle size did not cause significant variations in fatty acid content. Similar to the results of the present study, Hadbaoui et al. [29] reported a PUFA > MUFA > SFA pattern in sorghum grains. Results obtained for different fractions of Chinese barley showed that the highest total fatty acid content was found in the bran and germ fractions after steam heat treatment [30]. Due to their structure, amylose more easily forms complexes with MUFA [31]. Dry heat treatment has advantages over hydrothermal treatments in terms of increasing PUFA levels [32].

The most abundant fatty acids found in sorghum flours were palmitic acid, oleic + elaidic acid, linoleic + linoelaidic acid, and ɣ-linolenic acid (Table 1). Dry heat treatment determined the decrease in oleic + elaidic acid, linoleic + linoelaidic acid, and ɣ-linolenic acid, while the cis-8,11,14 eicosatrienoic + cis-11,14 eicosadienoic acid, henicosanoic acid, and eicosadienoic acid increased. Integral samples presented higher cis-8,11,14 eicosatrienoic + cis-11,14 eicosadienoic acid, henicosanoic acid, and eicosadienoic acid compared to the control and optimal fractions. To mitigate flavor instability and lipid rancidity in sorghum flour, rich in unsaturated fatty acids and highly susceptible to lipoxygenase-mediated oxidation, pre-milling heat treatment is essential for lipase inactivation and for enhancing shelf stability [11]. Another paper reported that white sorghum grains have 12.4% palmitic acid and >45% linoleic acid [29], which is in agreement with our results.

**Figure 3:**
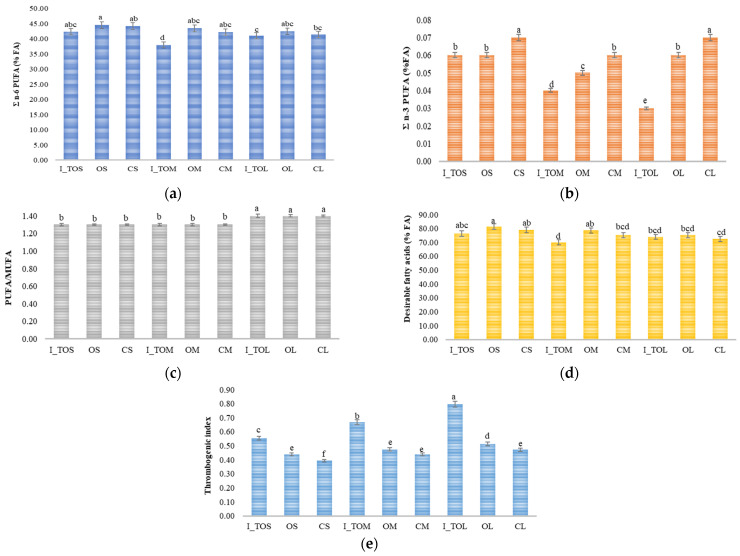
Variation in the content of polyunsaturated fatty acids omega-6 (n-6 PUFA) (**a**) and polyunsaturated fatty acids omega-3 (n-3 PUFA) (**b**), the ratio between polyunsaturated and monounsaturated fatty acids (PUFA/MUFA) (**c**), desirable fatty acids (DFA) (**d**), and the thrombogenic index (TI) (**e**) in integral white sorghum flour treated at the optimal temperature specific to each fraction, in the optimal, and control fractions: I_TOS—integral sorghum flour treated at 121 °C; I_TOM—integral sorghum flour treated at 132 °C; I_TOL—integral sorghum flour treated at 140 °C; CS—untreated control sample with S particle size (<200 μm); OS—optimal treated sample at 121 °C with S particle size (<200 μm); CM—untreated control sample with M particle size (200 μm < M < 250 μm); OM—optimal treated sample at 132 °C with M particle size (200 μm < M < 250 μm); CL—untreated control sample with L particle size (>300 μm); and OL—optimal treated sample at 140 °C with L particle size (>300 μm).

Figure 3a shows the variation in the total omega-6 polyunsaturated fatty acid (n-6 PUFA) content in the analyzed white sorghum flour samples. Higher values for n-6 PUFA are noted in the optimal fractions compared to the values obtained for the integral white sorghum flour samples. Compared to the control fractions (CL, CM, and CS), an increase in n-6 PUFA was obtained in the optimal fractions (OL, OM, and OS). The results indicated a slight decrease in the content of n-6 PUFA with the reduction in particle size. It has been shown that an increased intake of n-3/n-6 PUFA or MUFA considerably decreases the risk of cardiovascular disease, and there is evidence that replacing saturated fats with unsaturated fats is much more effective in reducing the risk of heart disease than reducing the amount of fat consumed [30]. It has been reported that during sorghum milling, lipase is released that degrades triglycerides, and consequently, the content of free fatty acids increases [1]. The variation in the content of omega-3 polyunsaturated fatty acids (n-3 PUFA) in the analyzed white sorghum flour samples is shown in Figure 3b. A significant decrease (*p* < 0.05) in the content of n-3 PUFA was observed in the optimal fractions after the application of heat treatment. Also, a remarkable decrease in the content of n-3 PUFA was obtained in the integral white sorghum flour treated at the optimal temperatures for each fraction (I_TOS, I_TOM, and I_TOL). Data on the influence of heat treatment and particle size of sorghum grains on the content of fatty acids in the specialized literature are limited. In rice flour, smaller particle fractions showed higher hydrophobicity and oil-binding ability than coarser fractions, and dry heat further increased these properties [33]. This indicates that finer particles are more affected by heat, suggesting that fatty acids in smaller fractions could possibly be more exposed and susceptible to thermal modification.

Figure 3c highlights different values for the ratio of polyunsaturated to monounsaturated fatty acids (PUFA/MUFA) in the analyzed samples. No significant differences (*p* > 0.05) were obtained between the heat-treated integral white sorghum flour samples, the optimal fractions, and the control. A slightly higher PUFA/MUFA ratio was noted in the optimal fraction L compared to the optimal fractions M and S. Similar results were reported by Paucar-Menacho [34] for expanded quinoa grains. The PUFA/MUFA ratio is a critical indicator of nutritional quality, as replacing saturated fats with unsaturated ones is associated with a reduced risk of cardiovascular disease [35]. However, the health impact of PUFAs is heavily dependent on the balance between n-6 and n-3 fatty acids [36]. In the analyzed sorghum samples, although the PUFA/MUFA ratio remained stable across most heat treatments, the inherent dominance of n-6 over n-3 fatty acids warrants consideration. While n-6 fatty acids are essential, a high n-6/n-3 ratio, often exceeding 15:1 in cereal-based diets has been linked to the promotion of systemic chronic inflammation, which contributes to the pathogenesis of obesity, type 2 diabetes, and metabolic syndrome [36]. Since the optimal dietary ratio is suggested to be closer to 4:1 or lower [36], the slight increase in PUFA/MUFA observed in fraction L, though minor, emphasizes the need to monitor the n-6/n-3 balance during processing. Heat treatments, like those applied to the sorghum fractions, must be carefully controlled to prevent the preferential oxidation of n-3 fatty acids, which are more chemically unstable than n-6, as this could further skew the ratio toward a pro-inflammatory profile [37].

The variation in the content of desirable fatty acids (DFA) in the analyzed white sorghum flour samples is represented in Figure 3d. Compared to the control fractions, it was observed that the dry heat treatment at the optimal fraction-specific temperature determined an increase in the DFA content in the optimal fractions. An increase in the DFA content was observed with the reduction in particle size, OS > OM > OL. The germ and the aleurone layer contain the highest percentage of fats [8]. Thus, the difference between the DFA content of the different fractions and compared to integral white sorghum flour can be explained.

The variation in the thrombogenic index (TI) in integral white sorghum flour heat-treated at the optimal temperature (I_TOS, I_TOM, and I_TOL) and in the optimal fractions (OS, OM, and OL), compared with fractions obtained from non-heat-treated grains (CS, CM, and CL), is presented in Figure 3e. TI is a compositional lipid-quality index derived from the fatty acid profile, introduced to better capture the theoretical thrombogenic potential of dietary lipids by weighting major pro-thrombogenic saturated fatty acids against anti-thrombogenic MUFA and PUFA classes; therefore, it should be interpreted as an indirect descriptor rather than a direct surrogate of clinical thrombosis risk [38].

Dry heat treatment increased the TI in all optimal fractions, although values were significantly lower (*p* < 0.05) than those of the heat-treated integral flour, while fractionation decreased the TI. Importantly, the observation that PUFA and MUFA increased while the TI also rose suggests that changes in specific SFA determinants (notably C14:0, C16:0, and C18:0) and/or shifts in PUFA subclasses (including the n-6/n-3 balance) may have outweighed the global increase in unsaturated fatty acids in the TI formula [39]. Any “lipid-related” implication should be contextualized by the absolute lipid content per serving and by potential heat-induced lipid oxidation, which may modify biological effects despite favorable changes in total PUFA/MUFA [40,41]. Epidemiological analyses have explored relationships between dietary atherogenic/thrombogenic indices and long-term cardiovascular outcomes, supporting the relevance of these indices as dietary descriptors, while still underscoring that they do not replace clinical endpoints [39,42]. Future work should therefore report the main SFA contributors driving the TI, the n-6/n-3 ratio, and lipid oxidation markers to strengthen translational interpretation when developing health-oriented sorghum-based products. The present study does not evaluate health endpoints, so the results should be interpreted as compositional characterization. Heat treatment increased PUFA/MUFA but also slightly increased the thrombogenic index, indicating a change in the relative balance between pro-thrombogenic and anti-thrombogenic fatty acids (as a compositional descriptor). In diabetes, early cardiovascular/autonomic dysfunction is a recognized concern and may be detectable via sudomotor markers [43].

### 2.3. Mineral Content

The mineral substances identified in the analyzed samples include calcium and sodium as macroelements, and iron, zinc, and copper as microelements (Table 2). The results obtained for the mineral content of white sorghum grains heat-treated at the optimal fraction-specific temperature (I_TOS, I_TOM, and I_TOL), from the optimal white sorghum flour fractions (OS, OM, and OL), compared to those obtained for the control fractions (CS, CM, and CL) indicate significant variations (*p* < 0.05) between samples. A decrease in calcium content was identified in the optimal fractions compared to the control fractions obtained from the fractionation of untreated white sorghum grains. Sodium content also decreased in the optimal fractions compared to the control, except for the optimal fraction S (OS), a fraction for which a high iron content was also obtained. The optimal fraction M (OM) is distinguished by an increase in iron, zinc and copper content compared to the control fraction (CM). Rashwan et al. [44] demonstrated that heat treatment increased the iron and zinc content by 20–44% and 4–29%, respectively, probably due to the degradation of the phytate-Fe and phytate-Zn complexes due to high temperature [44]. Viadel et al. [45] demonstrated that heat treatments affect the mineral content and their solubility, as well as the content of other components that can affect mineral solubility. Afify et al. [12] reported a lower iron, zinc, and copper content of sorghum grains after heat treatment.

According to a previous paper, pressure-cooking and microwave heating generally reduced zinc bioaccessibility in cereals and pulses by over 60% (depending on the type of grain), while iron bioaccessibility was significantly enhanced across all grains upon heat treatment [46]. It has been demonstrated that flaking and controlled decortication can enhance the in vitro mineral availability of sorghum by reducing tannin and phytate levels, highlighting the need for optimized sorghum variety selection, pre-treatments, and processing methods to maximize nutritional benefits [47]. Phytate-to-mineral molar ratios are key indicators of potential mineral bioavailability, with lower ratios corresponding to higher bioavailability and higher ratios indicating reduced mineral absorption [47]. Since heat treatment can reduce phytate content in sorghum grains [12], it can be presumed that an enhanced bioavailability of the minerals will be obtained. The mineral enrichment observed in component M may be explained by its microstructural characteristics and the distribution of grain components within the different particle sizes. The embryo and aleurone layers contain substantial phytate reserves, while the endosperm contains relatively lower levels of this compound [48]. Under heat conditions thermo-chemical and structural degradation of the matrix occurs [44], thereby releasing chelated minerals such as Fe and Zn and increasing their bioavailability.

### 2.4. Volatile Compounds

**Figure 4:**
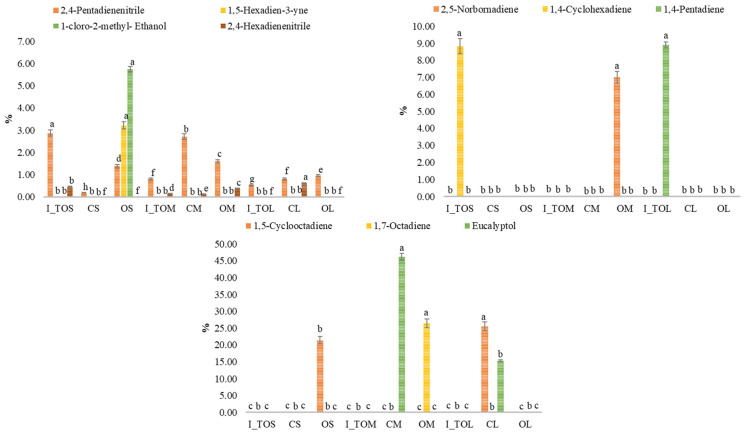
Volatile compounds from white sorghum flour treated at the optimal fraction-specific temperature, in the optimal, and control fractions: I_TOS—integral sorghum flour treated at 121 °C; I_TOM—integral sorghum flour treated at 132 °C; I_TOL—integral sorghum flour treated at 140 °C; CS—untreated control sample with S particle size (<200 μm); OS—optimal treated sample at 121 °C with S particle size (<200 μm); CM—untreated control sample with M particle size (200 μm < M < 250 μm); OM—optimal treated sample at 132 °C with M particle size (200 μm < M < 250 μm); CL—untreated control sample with L particle size (>300 μm); and OL—optimal treated sample at 140 °C with L particle size (>300 μm).

The variation in volatile compounds found in the sorghum flour samples is represented in Figure 4. Heat treatment determined the increase in the content of 2,4-pentadienenitrile in the optimal fraction L (OL) and its decrease in the optimal fraction M (OM).

The optimal fraction S (OS) was distinguished by the high concentration of 1-chloro-2-methyl-ethanol and 1,5-hexadiene, and the 2,4-pentadienenitrile compound identified in this optimal fraction was in a lower concentration compared to the concentration in the OM sample. The 2,5-norbornadiene was identified only in the optimal fraction M, not in the control fraction M, while 1,4-cyclohexadiene was present in the integral white sorghum flour treated at the optimal temperature of 121 °C, specific to the S fraction, and 1,4-pentadiene in the integral white sorghum flour treated at the optimal temperature of 140 °C, specific to the L fraction. The OM fraction is also distinguished by the compound 1,7-octadiene, a compound that was not identified in the control fraction CM. Similar to our study, Chen et al. [49] also reported significant amounts of terpenes (>25%) in sorghum flour. Fan et al. [50] demonstrated that the volatile profile of sorghum changed after cooking depending on the variety, as a result of the degradation of amino acids, lipid oxidation, and/or Maillard reactions.

In the optimal fraction S (OS), only 1,5-cyclooctadiene was identified, a compound that was also present in the control sample L (CL). The volatile compound eucalyptol, which was identified in the control fractions L and M (CL and CM) was no longer present in the optimal samples, indicating that the heat treatment had a negative effect on this compound. It can be mentioned that the dry heat treatment and fractionation influenced the volatile compound profile of sorghum. Among the three optimal fractions, the OS fraction was distinguished by a more complex volatile compound profile that included 1-chloro-2-methyl-ethanol, 1,5-hexadiene, 2,4-pentadienenitrile, and 1,5-cyclooctadiene. Nitrile compounds can be related to a bitter-almond aroma [51]. 1,4-Cyclohexadiene is a precursor of aromatic compounds and could give a herbaceous-citrusy aroma [52] to the sorghum flour. Eucalyptol has a minty, camphor-like, fresh odor [53]. Unsaturated hydrocarbons such as dienes and alkynes could be associated with a green, herbaceous, fatty, and galbanum-like odor, which is considered an undesirable flavor given by the oxidative breakdown of the unsaturated fats [49,54]. Thus, dry heat treatment can be suitable for modulating the sorghum flour aroma profile by reducing the number of undesirable compounds. In food products, volatile compounds from diverse chemical classes (terpenes, aldehydes, hydrocarbons, alcohols) are major determinants of aroma and consumer acceptance, supporting the importance of characterizing specific volatiles present in sorghum flour [55]. Eucalyptol, a monoterpenoid with a distinctive spicy aroma, can bring significant olfactory notes [53]; its presence in sorghum volatile profiles may alter aroma perception and hence acceptance. Unsaturated hydrocarbon volatiles and related compounds, though less commonly studied, can influence sensory perception. For example, higher concentrations of certain alkenes are linked with aromas that can detract from product acceptance in other food matrices [56].

### 2.5. Dynamic Rheological Parameters of White Sorghum Flour Dough

**Figure 5:**
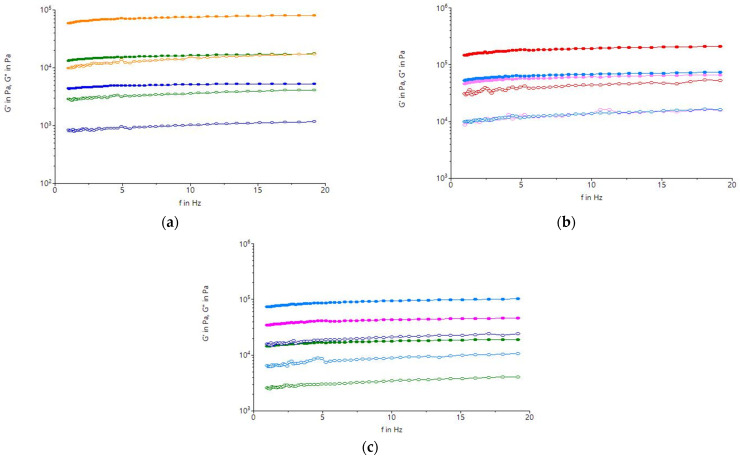
Frequency-dependent variation in the elastic modulus (G′, solid symbols) and viscous modulus (G″, open symbols) for samples with white sorghum flour treated at the following: (**a**) An optimum temperature of 121 °C, S particle size (<200 μm): I_TOS (-●-)—treated integral sorghum flour; OS (-●-)—optimal treated sample; and CS (-●-)—untreated control sample. (**b**) An optimum temperature of 132 °C, M particle size (200 μm < M < 250 μm): I_TOM (-●-)—treated integral sorghum flour; OM (-●-)—optimal treated sample; and CM (-●-)—untreated control sample. (**c**) An optimum tempearture of 140 °C, L particle size (>300 μm): I_TOL (-●-)—treated integral sorghum flour; OL (-●-)—optimal treated sample; and CL (-●-)—untreated control sample.

The elastic modulus (G′) and viscosity (G″) for the dough samples analyzed increased with frequency, indicating a more viscous behavior of the samples (Figure 5). The trends were fairly linear for all sorghum flour dough samples for the frequency range studied. Higher values of G′ and G″ moduli at higher frequencies suggest that more bonds are involved in the mechanical response of the system due to a stress or deformation applied in a shorter time. For all samples tested, the G′ modulus values were higher than the G″ modulus values, as expected for highly structured materials, indicating that the doughs were more elastic than viscous. The dough with the OL fraction had lower G′ values compared to the doughs made with the CL or with the integral sorghum flour I_TOL, which could probably be due to the lower amount of starch in the L fraction [2]. Also, probably the absorption of water by starch molecules tends to amplify the plasticizing effect of water. The decrease in G′ modulus for OL compared to CL indicated the considerable cumulative effect of dry heat treatment at 140 °C and fractionation on sorghum. The decrease in G′ value due to heat treatment was reported by Sharma et al. [57] who studied the effect of hydrothermal treatment on the rheological properties of pearl millet starch. In the case of dough with OM, the G′ modulus indicated a value slightly higher than that corresponding to the dough from I_TOM, but lower compared to the value corresponding to the dough with the CM (Figure 5). G′ was higher than G″ over the entire frequency range for all samples, which indicated a large difference between G′ and G″, suggesting their predominant elastic behavior [58]. The dough sample with OS showed a considerable increase in the modulus of elasticity (G′), while the sample with the CS showed the lowest values with increasing frequency. This high value could probably be due to the gelatinization of starch or the reduction in the plasticizing effect of water due to the absorption of water by starch molecules, which are found in higher amounts in the S particle size [2]. Significant differences in G′ values were also observed between the sample with the OS and that with integral I_TOS. The remarkable differences between the doughs with OL, OM, and OS regarding the moduli G′ and G″ could be explained by the chemical composition of these fractions (Table 1). Dry heat treatment applied to white sorghum grains may cause changes in the composition of sorghum flour fractions, with an impact on the rheological behavior of the dough. Poor viscoelastic properties for sorghum flour dough have been reported in a previous study [59].

**Figure 6:**
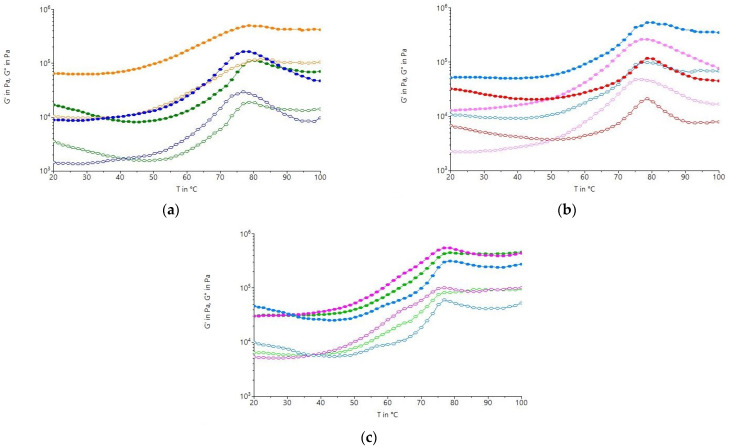
Temperature-dependent variation in the elastic modulus (G′, solid symbols) and viscous modulus (G″, open symbols) for samples with white sorghum flour treated at the following: (**a**) An optimal temperature of 121 °C, S particle size (<200 μm): I_TOS (-●-)—treated integral sorghum flour; OS (-●-)—optimal treated sample; and CS (-●-)—untreated control sample. (**b**) An optimal temperature of 132 °C, M particle size (200 μm < M < 250 μm): I_TOM (-●-)—treated integral sorghum flour; OM (-●-)—optimal treated sample; and CM (-●-)—untreated control sample. (**c**) An optimal temperature of 140 °C, L particle size (>300 μm): I_TOL (-●-)—treated integral sorghum flour; OL (-●-)—optimal treated sample; and CL (-●-)—untreated control sample.

The variation in the elastic modulus (G′) and viscosity (G″) with temperature indicated different values depending on the type of sample (Figure 6). At first, there is a minimum decrease in the G′ modulus up to a certain temperature due to proteins losing their ability to retain water; as the temperature increases, G′ increases sharply until the maximum starch gelatinization temperature is reached, and then decreases again due to starch degradation. In the case of the dough with the optimal L fraction (OL), the increase in temperature led to higher values for G′ and G″ compared to the values obtained for the doughs with the control L fraction (CL) and those with the integral white sorghum flour (I_TOL) treated at the optimal temperature of 140 °C. Dough with the optimal fraction M (OM) presented the lowest values for G′ and G″ with increasing temperature, and the heat treatment led to a decrease in the starch gelatinization temperature. Increasing the temperature above 65 °C resulted in a sudden increase in the G′ and G″ moduli, with the highest values of the moduli obtained for the dough with the optimal fraction S (OS). The OS presented higher values of the G′ and G″ moduli than those for the dough with the control fraction S (CS). Previous studies have reported a correlation between rheological parameters and water absorption [60], which may explain the behavior of the OS, as mentioned, due to the presence of gelatinized starch. For all the samples studied, the maximum gelatinization temperature varied depending on the thermal treatment temperature and the granulation of the white sorghum flour, ranging from 75 to 85 °C. The thermal treatment caused a decrease in the maximum gelatinization temperature, a decrease that was more pronounced in the OS and OM. Lower values of the maximum gelatinization temperature indicate a lower tendency to retrograde [61]. The decrease in maximum gelatinization temperature indicates the treatment caused partial disruption/plasticization of crystalline regions or promoted the formation of less stable crystallites that melt at a lower temperature [62]. In addition, thermal treatments can also cause partial leaching of amylose and the rearrangement of amorphous domains, changing the balance of crystalline vs. amorphous components and reducing the energy required for the remaining ordered regions to melt [63]. Smaller (S and M) granule fractions exhibit a more pronounced decrease in gelatinization temperature during thermal treatment compared to large granules, probably due to their larger surface-to-volume ratio, lower crystallinity, and more accessible amorphous regions [64,65].

**Figure 7:**
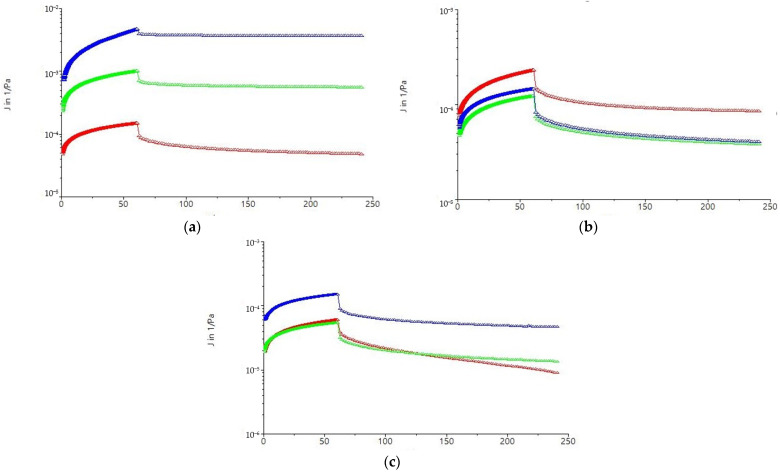
Creep and recovery curves for samples with white sorghum flour treated at the optimal temperature of: (**a**) 121 °C, (**b**) 132 °C, and (**c**) 140 °C: Integral (-●-), Optimal (-●-), and Control (-●-): I_TOS—integral sorghum flour treated at 121 °C; I_TOM—integral sorghum flour treated at 132 °C; I_TOL—integral sorghum flour treated at 140 °C; CS—untreated control sample with S particle size (<200 μm); OS—optimal treated sample at 121 °C with S particle size (<200 μm); CM—untreated control sample with M particle size (200 μm < M < 250 μm); OM—optimal treated sample at 132 °C with M particle size (200 μm < M < 250 μm); CL—untreated control sample with L particle size (>300 μm); and OL—optimal treated sample at 140 °C with L particle size (>300 μm).

Remarkable differences were observed for the maximum creep compliance (J_cmax_) and the maximum recovery compliance (J_rmax_) determined at the end of the creep and recovery phases, respectively, depending on the dough type (Figure 7). Dough deformation data can be used to evaluate dough strength, with a softer dough requiring less energy to achieve the same deformation compared to a harder dough [66]. The variations obtained for J_cmax_ and J_rmax_ can be explained by the starch or protein content of the sorghum flour types [2]. Hydroxyl groups in the starch composition that will form covalent and non-covalent bonds can cause a decrease in J_cmax_ and J_rmax_ values, suggesting a firm dough [67]. The maximum creep compliance value (J_cmax_) decreased significantly (*p* < 0.5) for the dough with the optimal L fraction (OL), which shows a lower resistance to deformation compared to the control sample (CL) [68]. The low-compliance dough sample resists deformation more strongly than the high-compliance sample, indicating the formation of strong bonds between the macromolecules of the optimal white sorghum flour fraction. The minimum value of J_cmax_ was obtained for the white sorghum integral flour dough I_TOL and indicates an increase in stiffness. The heat treatment caused a considerable decrease in the maximum rebound compliance, J_rmax_, of the dough with the optimal L fraction (OL). On the other hand, the OL showed a higher value for maximum compliance upon proofing (J_rmax_) compared to the value obtained for the dough from the integral white sorghum flour I_TOL, which suggests that the L particle size fractionation led to a less firm dough. The dough with the optimal M fraction (OM) presented lower values for maximum creep compliance (J_cmax_) and maximum recovery compliance (J_rmax_) (Figure 7), revealing an increase in strength and a lower capacity to deform and recover compared to the CM control sample. This could be due to interactions between dough compounds that would determine changes in macromolecular organization [69].

The maximum recovery compliance (J_rmax_) for the OM sample indicated a dough elasticity close to that corresponding to the sample with the control M fraction (CM). Considerable differences in creep and recovery were observed between the dough with the OM fraction and that made from the I_TOM. The maximum compliance obtained after 60 s of the creep test was recorded for the dough sample with the control fraction S (CS). This effect can be explained by the amount of water available in the dough sample from unheated white sorghum flour. The creep compliance increases at constant tension with increasing water content, while the contribution to elastic deformation decreases [70]. The OS showed the lowest values of maximum compliance (J_rmax_) compared to the dough from the CS and that from the I_TOS. The decrease in compliance values for the dough from the OS compared to the dough from the CS will lead to a decrease in dough extensibility. The maximum recovery compliance (J_rmax_) indicated the variation in dough elasticity depending on the type of sorghum flour sample. The dough with the OS fraction showed a higher value for J_rmax_ compared to the dough with the CS fraction, indicating an improvement in recovery behavior as a result of the heat treatment. Heat treatment may induce kafirin unfolding and disulfide-mediated aggregation that strengthens the protein network and, together with the formation of thermally stabilized amylose–lipid complexes, reduces starch swelling and enzyme access, leading to increased starch resistance and improved dough strength/elasticity [10,71,72]. S particle size samples were richer in oleic acid (Table 1), which has been demonstrated to form complexes with starch that are resistant to enzyme attack [72], possibly explaining the increased dough strength (lower compliances) compared to higher fractions. In addition, fractionation can determine the increase in damaged starch content, leading to increased amylose and free hydroxyl groups, which promote hydrogen bond formation between their glucose molecules and the glutamine-rich gluten protein, consequently altering dough properties [67]. Another study revealed that in barley flour, heat treatment by autoclaving increased dough elasticity, stiffness, and resistance to deformation, and these effects were more pronounced in coarse flour fractions compared with fine fractions, indicating that particle size modulates the impact of thermal processing on dough rheological properties [73]. Pre-milling thermal treatment of oat grains by kilning, combined with subsequent milling to different particle sizes, significantly affected dough rheology, including water absorption and extensibility, demonstrating that particle size modulates the impact of heat on the viscoelastic properties of oat flour dough [74].

In gluten-free dough systems, thermal treatments are known to increase dough elasticity and stiffness due to protein denaturation and partial starch gelatinization, leading to higher viscoelastic moduli (G′, G″) and reduced deformability under shear. This heat-induced rigidification of the dough network can enhance gas retention and expansion during proofing and baking, resulting in greater loaf volume and improved crumb structure compared with untreated systems [75]. However, if heat makes the dough too rigid, increased resistance to deformation can limit bubble expansion in the oven, reducing specific volume and yielding a denser crumb [76]. Thus, the observed decrease in deformability (lower creep compliance with G′ > G″) is directly linked to baking performance outcomes in gluten-free products, such as oven spring, texture, and crumb quality [75].

## 3. Materials and Methods

### 3.1. Materials

For the experimental research, white sorghum grains (*Sorghum bicolor* (L.) Moench ES Albanus) purchased from the Secuieni Agricultural Research and Development Station (Neamț, Romania) were used. The Agricultural Research and Development Station Secuieni is located in Neamț County, in the central Moldavian Plateau of northeastern Romania, at approximately 46°05′ N latitude and 26°05′ E longitude, in a humid continental climate characterized by cold winters, warm summers, and moderate precipitation [7]. The sorghum grains were processed by two methods, namely, they were first subjected to a dry heat treatment, and then they were ground. The dry heat treatment was applied to the sorghum grains placed on a tray in a uniform layer of 10 mm, in a convection oven (Binder ED53 L, BINDER GmbH, Tuttlingen, Germany), for 15 min, at different temperatures, 121 (for the S fraction), 132 (for the M fraction) and 140 °C (for the L fraction), according to the previous study that determined the optimal values for each fraction [2]. Our previous research established the optimal values for the treatment of each fraction based on the highest functional properties (e.g., water retention capacity, hydration capacity, swelling power, emulsifying characteristics) and nutrients content (protein, fat, ash), and the lowest carbohydrate content [2]. Integral flours (without fraction separations) treated at the three temperatures mentioned above were also studied. A KitchenAid grain mill (model 1065 KGM, Whirlpool Corporation, Benton Harbor, MI, USA) was used to grind the sorghum grains. The integral sorghum flour was then separated using a vibrating sieve system (Retsch AS, Retsch GmbH, Haan, Germany) into three types of granulations: large (L > 300 μm), medium (200 μm < M < 250 μm), and small (S < 200 μm).

The codification of the samples was the following: I_TOS—integral sorghum flour treated at 121 °C; I_TOM—integral sorghum flour treated at 132 °C; I_TOL—integral sorghum flour treated at 140 °C; CS—untreated control sample with S particle size (<200 μm); OS—optimal treated sample at 121 °C with S particle size (<200 μm); CM—untreated control sample with M particle size (200 μm < M < 250 μm); OM—optimal treated sample at 132 °C with M particle size (200 μm < M < 250 μm); CL—untreated control sample with L particle size (>300 μm); and OL—optimal treated sample at 140 °C with L particle size (>300 μm).

### 3.2. Determination of Amino Acids

Acid hydrolysis of sorghum flours was performed according to a modified protocol by Synaridou et al. [77]. Briefly, 5 g of the homogenized sample were hydrolyzed with 20 mL of 4 M HCl in sealed glass containers at 95 °C for 24 h. After cooling to room temperature, the hydrolysates were filtered through qualitative filter paper and neutralized with 15 mL of 10% (*w*/*v*) potassium hydroxide solution. The neutralized solutions were filtered again through 0.45 µm membrane filters before chromatographic analysis. Pre-column derivatization was carried out automatically by the autosampler using ortho-phthalaldehyde (OPA) reagent in a 1:2 sample-to-reagent ratio. The derivatization reaction was performed immediately before injection. Chromatographic analysis was performed using a Vanquish UHPLC system equipped with a fluorescence detector (Thermo Fisher Scientific, Waltham, MA, USA). Separation was achieved on a Hypersil Gold column (150 × 4.6 mm, 5 µm particle size) maintained at 25 °C. The mobile phase consisted of ultrapure water (A) and acetonitrile (B) applied in a gradient mode at a flow rate of 0.800 mL/min. The injection volume was 1 µL. Fluorescence detection was performed at an excitation wavelength of 340 nm and an emission wavelength of 450 nm. Quantification was performed using external calibration with an amino acid standard mixture. Calibration curves were constructed in appropriate concentration ranges for each amino acid, and linearity was accepted at R^2^ ≥ 0.995. All solvents were HPLC grade (VWR, Darmstadt, Germany), and ultrapure water was obtained using an Ultraclear UV UF Evoqua purification system (Evoqua Water Technologies GmbH, Pittsburgh, PA, USA). The OPA derivatization reagent (10 mg/mL o-phthalaldehyde and 3-mercaptopropionic acid in 0.4 M borate buffer) was purchased from Agilent (Santa Clara, CA, USA). The Amino Acid Standard mixture was obtained from Sigma-Aldrich (Saint Louis, MO, USA).

### 3.3. Determination of Fatty Acids

Fatty acid analysis was performed using a CG-DIF gas chromatograph (Agilent Technologies, 6890N GC, Wilmington, DE, USA) equipped with a DB-WAX capillary column with a polyethylene glycol stationary phase (30 m × 0.25 mm × 0.25 µm) and a flame ionization detector (FID). The samples (3 g) were extracted with 50 mL of chloroform:methanol (2:1, *v*/*v*) before their introduction into an ultrasonic bath (Sonorex RK 512H, BANDELIN electronic GmbH & Co. KG, Berlin, Germany). The extraction process was carried out in an ultrasonic bath for 30 min at room temperature (150 × 138 × 65 mm^3^, volume 1.3 L, ultrasonic power of 60 W, and a frequency of 40 kHz). After extraction, the samples were filtered, and the liquid fraction was recovered and extracted with 20 mL of KCl (0.74%). The extracts were subjected to a centrifugal process (10 min at 5000 rpm) to facilitate the separation of the organic phase. This phase was subsequently filtered using a solution of Na_2_SO_4_ to remove water. The solvent was then evaporated using a rotary evaporator (Laborota 4010, Heidolph Instruments GmbH & Co. KG, Schwabach, Germany), and the oil obtained was dried at 60 °C in an oven.

The standard FAME mixture (Supelco 37-component FAME mix, CRM47885) produced by Sigma-Aldrich (Merck KGaA, Darmstadt, Germany) was used as reference material. The results are expressed in relative percentage of each fatty acid, calculated by internal normalization of the chromatographic peak area.

Approximately 30 ± 0.1 mg of the resulting oil was dissolved in 2 mL of isooctane. The mixture was subjected to transesterification with 200 µL of methanolic potassium hydroxide solution (2 mol/L) under vigorous stirring. The resulting organic phase was mixed with sodium hydrogen sulfate. After collecting the supernatant, 1 µL of the sample was directly injected into the gas chromatograph (GC). The gases used for FID analysis were: hydrogen 40 mL/min, air 450 mL/min, and helium 30 mL/min. The injection volume was 1 µL in 1:20 split mode. The temperature program was as follows: the initial oven temperature was set at 60 °C, held for 1 min, increased from 60 to 200 °C by a 10 °C/min ramp, held for 2 min, and increased from 200 to 220 °C by a 5 °C/min ramp, then held for 20 min. The injector and detector temperatures were set at 250 °C. The identification of fatty acids in the samples was completed by comparing their retention times with those of the standard mixture [78].

The content of desirable fatty acids (DFA) was determined as the sum of MUFA, PUFA, and stearic acid [79].

The thrombogenic index was calculated as the sum of C14:0, C16:0 and C18:0 divided to (0.5 × MUFA + 0.5 × n − 6 + 3 × n − 3 + n3/n6) [80].

### 3.4. Determination of Minerals

The determination of mineral content was performed using a Shimadzu AA7000, ASC-7000 (Shimadzu Corporation, Kyoto, Japan) atomic absorption spectrophotometer (air-acetylene) equipped with an ASC-6100 autosampler (Shimadzu Corporation, Kyoto, Japan). The mineral content profile was determined for Ca, Na, Fe, Cu and Zn, elements that are predominant in sorghum flour. The samples were prepared according to the method described in the technical book of the Shimadzu AA7000, ASC-7000 spectrophotometer. A 5 g sample was placed in the crucible and calcined at 550 °C for 12 h. If calcination was incomplete, 1 mL of 65% nitric acid (from Merck KGaA, Darmstadt, Germany) was added to the crucible, and after the nitric acid evaporated, the calcination process was resumed until the white color of the ash appeared. After cooling, 1 mL of 65% nitric acid was added to the crucible, and the contents were transferred to a 50 mL volumetric flask and made up to the mark with deionized water [81]. Calibration curves were obtained using standard solutions (from Sigma Aldrich, Merck KGaA, Darmstadt, Germany) for each element (R^2^ > 0.99). The specific wavelengths for Ca, Na, Fe, Cu, and Zn were 422.7, 589.0, 248.3, 324.8, and 213.9 nm, respectively.

### 3.5. Determination of Volatile Compounds

The determination of volatile organic compounds was performed using a gas chromatograph coupled to a mass spectrometer (GC-MS 6890N, Agilent Technologies, Santa Clara, CA, USA), equipped with a HP-5-MS capillary column (60 m length, 0.2 mm I.D., and 0.25 µm film thickness (Agilent Technologies, Santa Clara, CA, USA). A 3 g sample was transferred to a 20 mL headspace vial, and 3 g sodium chloride was added to enhance the volatile organic compounds in the headspace and inhibit any enzymatic reaction. The headspace vials were sealed with crimp-top caps with TFE-silicone headspace septa. The criteria for compound identification required a mass spectral match score of ≥ 80%. The results were expressed as the percentage of relative peak area (%RPA) of a peak in each sample, which was calculated by dividing the peak area by the total area of all peaks identified in each chromatogram. The total ion chromatogram (TIC) of each sample was used for peak area integration. All measurements were performed in triplicate, and data are presented as mean ± standard deviation. The GC oven temperature was set at 35 °C (held for 1 min), increased to 100 °C (held for 1 min) at a rate of 5 °C/min, then to 250 °C (held for 3 min) at a rate of 7 °C/min, and finally increased to 250 °C (held for 1 min) at a rate of 10 °C/min. The transfer line temperature was set at 280 °C, and the ion source temperature was set at 250 °C. The mass spectrometer was operated in electron ionization (EI) mode at 70 eV according to the method reported by Dippong et al. [82].

### 3.6. Determination of Rheological Properties

The rheological properties of sorghum flour dough were determined by applying dynamic testing methods such as oscillatory testing and creep and recovery testing. A preliminary test was also applied to identify the limits of the linear viscoelastic region of the samples, at a constant oscillatory frequency of 1 Hz, by increasing the shear stress from 0 to 100 Pa. Dynamic rheological measurements were performed with a Thermo-HAAKE MARS 40 (Thermo Scientific, Karlsruhe, Germany) parallel plate dynamic rheometer, equipped with a Peltier heating system that allowed the sample to be maintained at a constant temperature (21 °C) during testing. The dough samples, prepared from 50 g of sorghum flour and 50 mL of water (55% moisture) mixed for 5 min, were left to stand for 5 min before testing to reach equilibrium. During testing, the distance between the plates was set to 2 mm, excess dough was removed, and to avoid moisture loss during testing of the sample, a layer of petroleum jelly was applied to its edge. Data processing was performed using the RheoWin Data Manager version 4.85 program (Thermo Scientific).

The determination of the variation in the moduli with frequency was carried out by applying a constant strain of 10 Pa, located in the linear viscoelastic region, in the frequency range from 0.01 to 20 Hz. The shear stress of 10 Pa was selected because it was in the linear segment range of the G′, G″–shear stress scan curve. An oscillatory test at a constant frequency of 1 Hz, in which the dough was heated at a rate of 4 ± 0.1 °C/min from a temperature of 20 to 100 °C was performed, and the values for the elastic and viscous moduli were registered. The dough was tested for creep and recovery by suddenly applying a voltage of 50 Pa (located in the linear viscoelastic range) for 60 s, and after removing the strain, it was left at rest for 180 s for recovery [83].

### 3.7. Statistical Analysis

The measurements were done in triplicate, and mean values with standard deviations were reported. The differences between samples were established by using ANOVA with Tukey’s test, at a significance level of 95%. All the statistical processing of data was done using XL STAT software (2020 and 2024 versions for Excel (Addinsoft, New York, NY, USA)).

## 4. Conclusions

Based on the findings, the application of dry heat treatment to white sorghum flour induces significant, particle-size-dependent changes across its chemical and mechanical properties. The findings describe composition and lipid-quality indices without implying clinical health effects. The treatment favorably modified the lipid profile by increasing PUFA and MUFA while reducing SFA, with the smallest fraction (S < 200 μm) exhibiting the optimal fatty acid balance. Simultaneously, the heat processing altered protein composition (e.g., lower lysine) and mineral content (e.g., lower calcium), while generating a unique volatile compound signature in the S fraction. The amino acid and fatty acid profiles of white sorghum flour fractions indicated that fractionation and heat treatment can enhance branched-chain amino acid content and desirable unsaturated fatty acids, supporting potential benefits for muscle protein synthesis and cardiometabolic health, yet these effects must be interpreted within the context of overall protein quality, lysine limitation, PUFA balance, and potential heat-induced modifications, emphasizing the importance of dietary context and food formulation in translating these compositional changes into meaningful clinical outcomes. From a rheological point of view, the heat treatment resulted in a stiffer, less deformable dough (lower creep compliance) that maintained elastic dominance (G′ > G″) across all fractions, coupled with a slight reduction in peak gelatinization temperature. Overall, these modifications demonstrate that thermal processing combined with controlled fractionation is an effective method for tailoring the functional and nutritional quality of sorghum flour for specific applications. Because the focus of this work was on comparing heat-treated whole-mill and graded flours, the unprocessed and ungraded whole flour was not included as a baseline control; therefore, the results primarily support relative comparisons between heat-treated samples rather than absolute changes from the raw material, and the conclusions are limited to the relative effects of particle grading under identical thermal conditions. Further research will be directed to study the applications of treated sorghum flour in food products and oxidative stability tests.

## Figures and Tables

**Table 1:** Fatty acid profile of sorghum flours (% total fatty acids).

Sample	I_TOS	CS	OS	I_TOM	CM	OM	I_TOL	CL	OL
Caprilic acid	0.32 ± 0.01 ^c^	0.02 ± 0.00 ^g^	0.12 ± 0.00 ^f^	0.33 ± 0.01 ^c^	0.03 ± 0.00 ^g^	0.14 ± 0.00 ^e^	0.35 ± 0.01 ^b^	0.50 ± 0.01 ^a^	0.16 ± 0.00 ^d^
Capric acid	0.11 ± 0.00 ^b^	0.05 ± 0.00 ^g^	0.10 ± 0.00 ^c^	0.10 ± 0.00 ^c^	0.04 ± 0.00 ^h^	0.07 ± 0.00 ^e^	0.13 ± 0.00 ^a^	0.06 ± 0.00 ^f^	0.08 ± 0.00 ^d^
Myristic acid	0.17 ± 0.00 ^a^	0.05 ± 0.00 ^e^	0.12 ± 0.00 ^c^	0.16 ± 0.00 ^b^	0.03 ± 0.00 ^f^	0.11 ± 0.00 ^d^	0.16 ± 0.00 ^b^	0.05 ± 0.00 ^e^	0.12 ± 0.00 ^c^
Myristoleic acid	0.22 ± 0.01 ^a^	0.02 ± 0.00 ^e^	0.05 ± 0.00 ^c^	0.22 ± 0.01 ^a^	0.06 ± 0.00 ^b^	0.05 ± 0.00 ^c^	0.22 ± 0.01 ^a^	0.05 ± 0.00 ^c^	0.04 ± 0.00 ^d^
Pentadecanoic acid	0.55 ± 0.01 ^a^	0.04 ± 0.00 ^d^	0.35 ± 0.01 ^b^	0.54 ± 0.01 ^a^	0.05 ± 0.00 ^d^	0.35 ± 0.01 ^b^	0.55 ± 0.01 ^a^	0.06 ± 0.00 ^d^	0.32 ± 0.01 ^c^
Plamitic acid	18.20 ± 0.45 ^c^	14.2 ± 0.36 ^g^	16.20 ± 0.41 ^def^	20.10 ± 0.50 ^b^	15.20 ± 0.38 ^fg^	16.50 ± 0.41 ^de^	25.60 ± 0.64 ^a^	15.68 ± 0.39 ^ef^	17.20 ± 0.43 ^cd^
Palmitoleic acid	0.65 ± 0.02 ^cd^	0.58 ± 0.01 ^e^	0.68 ± 0.02 ^c^	0.62 ± 0.02 ^de^	0.42 ± 0.01 ^f^	0.77 ± 0.02 ^b^	0.85 ± 0.02 ^a^	0.44 ± 0.01 ^f^	0.74 ± 0.02 ^b^
Heptadecanoic acid	0.30 ± 0.01 ^bc^	0.11 ± 0.00 ^e^	0.28 ± 0.01 ^d^	0.31 ± 0.01 ^b^	0.12 ± 0.00 ^e^	0.29 ± 0.01 ^cd^	0.34 ± 0.01 ^a^	0.12 ± 0.00 ^e^	0.29 ± 0.01 ^cd^
Cis-10-heptadecenoic acid	0.21 ± 0.01 ^c^	0.12 ± 0.00 ^e^	0.15 ± 0.00 ^d^	0.23 ± 0.01 ^b^	0.11 ± 0.00 ^e^	0.16 ± 0.00 ^d^	0.25 ± 0.01 ^a^	0.12 ± 0.00 ^e^	0.20 ± 0.01 ^c^
Stearic acid	2.2 ± 0.06 ^c^	1.15 ± 0.03 ^f^	1.33 ± 0.03 ^e^	2.40 ± 0.06 ^b^	1.08 ± 0.03 ^f^	1.65 ± 0.04 ^d^	2.70 ± 0.07 ^a^	1.15 ± 0.03 ^f^	1.62 ± 0.04 ^d^
Oleic + elaidic acid	30.4 ± 0.76 ^bcd^	34.52 ± 0.86 ^a^	32.52 ± 0.81 ^ab^	28.30 ± 0.71 ^de^	32.52 ± 0.81 ^ab^	30.56 ± 0.76 ^bc^	26.50 ± 0.66 ^e^	30.25 ± 0.76 ^cd^	28.56 ± 0.71 ^cde^
Linoleic + linoelaidic acid	39.6 ± 0.99 ^ab^	42.30 ± 1.06 ^a^	41.5 ± 1.04 ^a^	35.50 ± 0.89 ^c^	41.26 ± 1.03 ^ab^	39.56 ± 0.99 ^ab^	34.20 ± 0.85 ^c^	40.26 ± 1.01 ^ab^	38.50 ± 0.96 ^b^
ɣ-linolenic acid	1.89 ± 0.05 ^d^	2.21 ± 0.06 ^ab^	2.10 ± 0.05 ^bc^	1.62 ± 0.04 ^e^	2.20 ± 0.06 ^ab^	2.00 ± 0.05 ^cd^	1.52 ± 0.04 ^e^	2.18 ± 0.05 ^ab^	2.30 ± 0.06 ^a^
α-linolenic acid	0.06 ± 0.00 ^b^	0.06 ± 0.00 ^b^	0.07 ± 0.00 ^a^	0.04 ± 0.00 ^d^	0.05 ± 0.00 ^c^	0.06 ± 0.00 ^b^	0.02 ± 0.00 ^e^	0.06 ± 0.00 ^b^	0.07 ± 0.00 ^a^
Arachidic acid	0.24 ± 0.01 ^a^	0.13 ± 0.00 ^e^	0.20 ± 0.01 ^b^	0.23 ± 0.01 ^a^	0.10 ± 0.00 ^f^	0.15 ± 0.00 ^d^	0.24 ± 0.01 ^a^	0.15 ± 0.00 ^d^	0.17 ± 0.00 ^c^
11-eicosenoic acid	0.25 ± 0.01 ^b^	0.22 ± 0.01 ^cd^	0.23 ± 0.01 ^c^	0.26 ± 0.01 ^ab^	0.21 ± 0.01 ^d^	0.22 ± 0.01 ^cd^	0.27 ± 0.01 ^a^	0.18 ± 0.00 ^e^	0.25 ± 0.01 ^b^
Cis-8,11,14 eicosatrienoic + Cis-11,14 eicosadienoic acid	0.83 ± 0.02 ^a^	0.03 ± 0.00 ^c^	0.63 ± 0.02 ^b^	0.85 ± 0.02 ^a^	0.05 ± 0.00 ^c^	0.65 ± 0.02 ^b^	0.82 ± 0.02 ^a^	0.04 ± 0.00 ^c^	0.64 ± 0.02 ^b^
Henicosanoic acid	0.55 ± 0.01 ^a^	0.24 ± 0.01 ^c^	0.35 ± 0.01 ^b^	0.55 ± 0.01 ^a^	0.24 ± 0.01 ^c^	0.35 ± 0.01 ^b^	0.54 ± 0.01 ^a^	0.25 ± 0.01 ^c^	0.36 ± 0.01 ^b^
Eicosadienoic acid	0.21 ± 0.01 ^b^	0.09 ± 0.00 ^ef^	0.11 ± 0.00 ^d^	0.22 ± 0.01 ^ab^	0.08 ± 0.00 ^f^	0.13 ± 0.01 ^c^	0.23 ± 0.01 ^a^	0.09 ± 0.00 ^ef^	0.10 ± 0.00 ^de^

a–h—small letters which are different within the same column indicate significant statistical differences (*p* < 0.05) between samples. I_TOS—integral sorghum flour treated at 121 °C; I_TOM—integral sorghum flour treated at 132 °C; I_TOL—integral sorghum flour treated at 140 °C; CS—untreated control sample with S particle size (<200 μm); OS—optimal treated sample at 121 °C with S particle size (<200 μm); CM—untreated control sample with M particle size (200 μm < M < 250 μm); OM—optimal treated sample at 132 °C with M particle size (200 μm < M < 250 μm); CL—untreated control sample with L particle size (>300 μm); OL—optimal treated sample at 140 °C with L particle size (>300 μm).

**Table 2:** Macro- and microelements in integral white sorghum flour treated at the optimal temperature specific to each fraction (I_TOS, I_TOM and I_TOL), in the optimal (OS, OM, and OL) and control fractions (CS, CM, and CL).

Sample	Ca (mg/kg)	Na (mg/kg)	Fe (mg/kg)	Zn (mg/kg)	Cu (mg/kg)
I_TOS	13.81 ± 0.35 ^c^	60.11 ± 1.50 ^bc^	29.67 ± 0.74 ^f^	11.88 ± 0.30 ^e^	2.82 ± 0.07 ^c^
OS	11.31 ± 0.28 ^d^	68.34 ± 1.71 ^b^	57.22 ± 1.43 ^b^	17.48 ± 0.44 ^a^	3.47 ± 0.09 ^a^
CS	12.42 ± 0.31 ^e^	63.00 ± 1.58 ^a^	52.23 ± 1.31 ^a^	18.97 ± 0.47 ^b^	3.84 ± 0.10 ^b^
I_TOM	13.67 ± 0.34 ^c^	64.39 ± 1.61 ^ab^	43.74 ± 1.09 ^d^	17.94 ± 0.45 ^ab^	2.41 ± 0.06 ^d^
OM	13.98 ± 0.35 ^bc^	57.93 ± 1.45 ^c^	55.72 ± 1.39 ^a^	18.09 ± 0.45 ^ab^	3.33 ± 0.08 ^b^
CM	17.50 ± 0.44 ^a^	62.22 ± 1.56 ^bc^	40.59 ± 1.01 ^d^	15.95 ± 0.40 ^c^	2.94 ± 0.07 ^c^
I_TOL	14.82 ± 0.37 ^b^	62.07 ± 1.55 ^bc^	36.24 ± 0.91 ^e^	13.38 ± 0.33 ^d^	1.16 ± 0.03 ^e^
OL	13.81 ± 0.35 ^c^	63.79 ± 1.59 ^b^	48.19 ± 1.20 ^c^	17.05 ± 0.43 ^bc^	2.84 ± 0.07 ^c^
CL	14.45 ± 0.36 ^bc^	68.45 ± 1.71 ^a^	47.76 ± 1.19 ^c^	17.62 ± 0.44 ^b^	3.75 ± 0.09 ^a^

a–f—small letters which are different within the same column indicate significant statistical differences (*p* < 0.05) between samples. I_TOS—integral sorghum flour treated at 121 °C; I_TOM—integral sorghum flour treated at 132 °C; I_TOL—integral sorghum flour treated at 140 °C; CS—untreated control sample with S particle size (<200 μm); OS—optimal treated sample at 121 °C with S particle size (<200 μm); CM—untreated control sample with M particle size (200 μm < M < 250 μm); OM—optimal treated sample at 132 °C with M particle size (200 μm < M < 250 μm); CL—untreated control sample with L particle size (>300 μm); OL—optimal treated sample at 140 °C with L particle size (>300 μm).

## Data Availability

Data will be available on request.
